# Flaxseed orbitides, linusorbs, inhibit LPS-induced THP-1 macrophage inflammation

**DOI:** 10.1039/c9ra09058d

**Published:** 2020-06-15

**Authors:** Xian-Guo Zou, Youn Young Shim, Jae Youl Cho, Deok Jeong, Jian Yang, Ze-Yuan Deng, Martin J. T. Reaney

**Affiliations:** College of Food Science and Technology, Zhejiang University of Technology Hangzhou 310014 Zhejiang China; State Key Laboratory of Food Science and Technology, Nanchang University Nanchang Jiangxi 330047 China dengzy@ncu.edu.cn; Department of Plant Sciences, University of Saskatchewan Saskatoon Saskatchewan S7N 5A8 Canada martin.reaney@usask.ca +1 306 966-5015; Prairie Tide Diversified Inc. Saskatoon Saskatchewan S7J 0R1 Canada; Guangdong Saskatchewan Oilseed Joint Laboratory, Department of Food Science and Engineering, Jinan University Guangzhou Guangdong 510632 China; Department of Integrative Biotechnology, College of Biotechnology and Bioengineering, Sungkyunkwan University Suwon Gyeonggi-do 16419 Korea; Drug Discovery and Development Research Group, College of Pharmacy and Nutrition, University of Saskatchewan Saskatoon Saskatchewan S7N 5E5 Canada

## Abstract

Linusorbs (flaxseed orbitides) are a family of naturally-occurring cyclic peptides. Previously, we reported that their anticancer effects were associated with their structures. In this study, we investigated the anti-inflammatory activities of 2 different linusorbs ([1–9-NαC]-linusorb B2 and [1–9-NαC]-linusorb B3) in lipopolysaccharide (LPS)-induced THP-1 macrophage activation as well as the underlying mechanism of this inflammatory response. Both molecules suppressed pro-inflammatory mediators (TNF-α, IL-1β, IL-6, NO and COX-2) and were involved in downregulating the NF-κB signaling pathway. The suppressive effects on pro-inflammatory mediators were comparable and the concentration range of action was similar (1–4 μM). However, the concentration of compound that induced downregulation of the NF-κB pathway was different for each compound. While [1–9-NαC]-linusorb B3 could inhibit the activation of the NF-κB pathway at concentrations of 1 and 2 μM, [1–9-NαC]-linusorb B2 induced a comparable inhibitory effect at a concentration of 4 μM.

## Abstract 2

Linusorbs (flax orbitides) are a family of plant cyclic peptides. We investigate the anti-inflammatory activities of two different linusorbs ([1–9-NaC]-linusorb B2 and [1–9-NaC]-linusorb B3) and the underlying mechanism of this inflammatory response.

## Graphical abstract

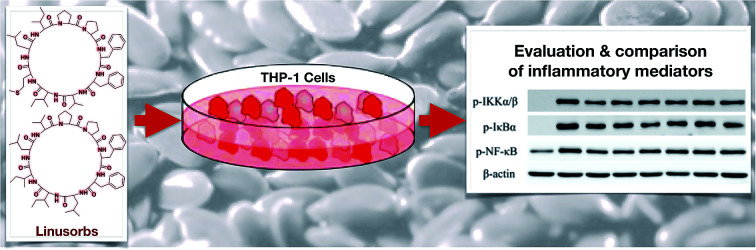

## 1. Introduction

Inflammation is an important host immune response to diverse stimuli, including physical injury, infections by pathogenic bacteria and viruses, tissue damage and toxins. However, excessive inflammation can result in uncontrolled production of inflammatory mediators, such as cytokines, eicosanoids, chemokines, reactive oxygen species (ROS) and adhesion molecules, leading to increased morbidity from chronic diseases, such as cardiovascular disease,^1^ type 2 diabetes,^2^ obesity,^3^ inflammatory bowel disease^4^ and cancer.^5^ Dietary interventions that control the release of pro-inflammatory mediators might prevent and/or cure the aforementioned diseases. However, prolonged consumption of synthetic drugs to suppress inflammation can cause adverse side effects. It has been reported that non-steroidal anti-inflammatory drugs (NSAIDs) including non-selective NSAIDs (nsNSAIDs), coxibs and glucocorticoids can exert deleterious effects on the cardiovascular system, leading to an increased risk of cardiovascular disease.^6–8^ Thus, there is an urgent need for development of alternative and safer therapeutic options based on natural compounds or their derivatives to alleviate inflammatory metabolic disorders.

Many *in vitro* cell studies and *in vivo* animal studies demonstrated that bioactive peptides from foods derived from fungi, animals and plants have anti-inflammatory effects. For example, Zhang *et al.*^9^ reported that two peptides, γ-glutamyl cysteine and γ-glutamyl valine, reduced the concentration of pro-inflammatory cytokines, including interleukin (IL)-8, IL-6, and IL-1β, in tumor necrosis factor alpha (TNF-α) stimulated Caco-2 cells. In the same study these peptides ameliorated weight loss, colon shortening and histological damage while increasing production of cytokines in a mouse colitis model. Similarly, the cyclic peptide citrusin X, purified from *Citrus unshiu* fruit, was reported to have anti-inflammatory activity by suppressing pro-inflammatory mediator production.^10^ Increasingly it is becoming apparent that complex cyclic peptides in extracts constitute a unique class of anti-inflammatory metabolites.

Linusorbs (LOs, flaxseed orbitides), are bioactive eight or nine amino acid N- to C-linked cyclic peptides present in flax (*Linum usitatissimum* L.).^11^ Kaneda *et al.*^12^ reported that LOs and their derivatives could retard proliferation of osteoclast cells. Shim and Reaney^13^ studied the binding abilities of LOs to human serum albumin, showing that [1–9-NαC]-linusorb B3 (LOB3) had about 2.5-fold higher binding than [1–9-NαC]-linusorb B2 (LOB2). Cho *et al.*^14^ reported a pharmaceutical composition for preventing or treating inflammatory diseases, consisting of LO mixture (LOMIX) derived from flaxseed as an active ingredient. Moreover, two LOs exhibited anticancer effects against SGC-7901 cells by inducing cell apoptosis and blocking cell cycle in G1 phase with the involvement of different signaling pathways.^15,16^ Nevertheless, there is little knowledge of LOs anti-inflammatory activities and the molecular mechanisms involved in such activity.

The mechanisms that trigger inflammation are usually shared, for example, the NF-κB signaling pathway is activated rapidly upon stimulation by a number of factors. The most prevalent form of NF-κB in normal cells, the p50/p65 dimer, is sequestered in the cytoplasm through binding with its inhibitor, IκB protein. Once the cell is stimulated, IκB is phosphorylated by specific IκB-kinases complex (IKK) and degraded rapidly leading to release of NF-κB.^17^ Free p65, one of the most active forms of NF-κB subunit, can translocate to the nucleus where it regulates gene expression and secretion of inflammatory cytokines.^18^ Numerous reports indicate that the anti-inflammatory effects of bioactive compounds or pharmacological drugs are associated with suppression of the NF-κB signaling pathway.^19–21^

The aim of this study was to explore the inflammatory activities of 2 LOs (LOB2 and LOB3) and examine the potential mechanism of their involvement in modifying the NF-κB pathway using THP-1 macrophages as a representative cell model.

## 2. Experimental

### 2.1. Materials and chemicals

LOB2 and LOB3 were provided as an in-kind contribution from Prairie Tide Diversified Inc. (PTD, Saskatoon, SK Canada). The human THP-1 monocytic cell line was obtained from American Type Culture Collection (ATCC, Rockville, MD, USA). Roswell Park Memorial Institute (RPMI)-1640 supplemented with l-glutamine and HEPES, fetal bovine serum (FBS), and penicillin–streptomycin (P/S, 10 000 IU mL^−1^) were purchased from Gibco BRL (Life Technologies Ltd., Paisley, UK). Lipopolysaccharide (LPS), 3-(4,5-dimethylthiazol-2-yl)-2,5-diphenyltetrazolium bromide (MTT), dimethylsulfoxide (DMSO), phosphate buffered saline solution (PBS), 2-mercaptoethanol, phorbol 12-myristate 13-acetate (PMA), and other chemicals were of analytical grade and obtained from Sigma-Aldrich (St. Louis, MO, USA). Protease and phosphatase inhibitor cocktail, radio immunoprecipitation assay (RIPA) lysis buffer, BCA protein assay kit, Griess reagent kit and instant enzyme-linked immunosorbent assay (ELISA) kits of human IL-1β, IL-6 and TNF-α were from Thermo Fisher Scientific (Waltham, MA, USA). Primary antibodies against β-actin, cyclooxygenase-2 (COX-2), p-IKKα/β, p-IκBα and p-p65-NF-κB were obtained from Cell Signaling Technology (Danvers, MA, USA).

### 2.2 Cell culture and treatment

THP-1 cells were routinely cultured in RPMI-1640 medium supplemented with 10% FBS and 1% P/S at 37 °C in a humidified atmosphere containing 5% carbon dioxide. Medium was replaced every 2 days and cells were passaged when reaching a density of 1 × 10^6^ cells per mL. Monocyte-macrophage differentiation was induced in THP-1 cells by treating with 100 ng mL^−1^ PMA for 48 h and then culturing them in fresh RPMI-1640 for 24 h prior to experimental use. In all experiments, THP-1 cells were seeded into plates at a density of 5 × 10^5^ cells per mL and the differentiated macrophages were incubated with LOB2 or LOB3 which was added to the medium for 1 h before LPS treatment (1 μg mL^−1^ LPS for 3 h). Equal volumes of PBS were added to the media of treatment and control cells. Cells used in this study were between passages 30 and 50.

### 2.3 Cell viability assay

Cytotoxic effects of LOs on cells were determined by an MTT assay. In brief, macrophages (1 × 10^5^ per well) were pretreated with 200 μL per well of various concentrations of LOB2 or LOB3 (0, 0.5, 5, 10, 20, 50, 100 and 200 μM) for 1 h. Subsequently, they were treated with LPS (1 μg mL^−1^) for 3 h. To test viability after treatment, 0.10 mL of 10% MTT solution (5 mg mL^−1^) was added to each well and cells were incubated for 4 h in the dark. The media were then discarded, and 0.10 mL of DMSO was added to dissolve precipitated formazan with gentle shaking for 10 min. Absorbance (OD) was measured at 490 nm with a microplate reader (SpectraMax 190, Molecular Devices, Sunnyvale, CA, USA).

### 2.4 Measurement of total nitrite by Griess reaction

Production of nitric oxide (NO) was determined from the total nitrite in the media. Macrophages (5 × 10^5^ cells per mL) were placed in 24-well plates and pretreated with different concentrations of LOB2 or LOB3 (0, 0.5, 1, 2, 4 and 8 μM) for 1 h and then, after 1 h, with 1 μg mL^−1^ LPS for 3 h. After treatment, each culture supernatant of 50 μL was mixed with Griess reagent and the absorbance at 550 nm was determined using a microplate reader. Finally, the NO production in each sample was quantified according to a standard curve of sodium nitrite.

### 2.5 ELISA assay for cytokine determination

Macrophages (THP-1; 5 × 10^5^ cells per mL) were plated in 24-well plates and treated with various concentrations of LOB2 or LOB3 (0, 0.5, 1, 2, 4 and 8 μM) for 1 h and then with LPS (1 μg mL^−1^) for 3 h. Supernatants were collected and concentrations of TNF-α, IL-1β and IL-6 were determined using corresponding ELISA kits according to manufactures' instructions.

### 2.6 Preparation of whole-cell lysate for western blot analysis

Macrophages (THP-1) were plated in 60-mm dishes and treated with various concentrations of LOB2 or LOB3 (0, 1, 2 and 4 μM) for 1 h prior to stimulation with 1 μg mL^−1^ LPS for 3 h. After cleaning the macrophages twice with cold PBS, cells were lysed for 15 min with 500 μL ice-cold RIPA buffer with protease and phosphatase inhibitor cocktail added. After incubation, the lysates were collected and centrifuged at 13 000 g for 15 min, and supernatants were collected as whole-cell lysates and stored at −80 °C until used.

### 2.7 Western blot analysis for determination of inflammatory associated proteins

Western blot assays were conducted according to our recent study.^16^ Protein concentrations were determined using a BCA protein assay kit (Pierce, Rockford, IL, USA). Subsequently, protein of each sample was mixed with 2× loading buffer and denatured by boiling for 5 min. Protein lysates (40 μg) were loaded onto and separated by 10% SDS-PAGE, then transferred to PVDF membranes. After blocking with 5% nonfat milk containing 1% Tween-20 (v/v) at room temperature for 1 h the membranes were then incubated with primary specific antibodies of anti-(β-actin, COX-2, p-p65NFκB, p-IκBα and p-IKKα/β) overnight at 4 °C. Following incubation with specific antibodies, appropriate secondary antibodies linked to horseradish peroxidase were added at room temperature for 1 h. Finally, protein bands were visualized and imaged using an enhanced chemiluminescence detection system (ECL) from Amersham Biosciences Corp. (Piscataway, NJ, USA).

### 2.8 Statistical analysis

All experiments in this study were conducted at least three times and data were reported as mean ± standard deviation. Differences among treatments and controls were analyzed using the Student's *t*-test as embodied in SPSS 19.0 software. A *p* value of <0.05 (*), <0.01 (**), and <0.001 (***) were considered statistically significant.

## 3. Results

### 3.1 Effects of LOB2 or LOB3 on THP-1 macrophage viability

**Fig. 1:**
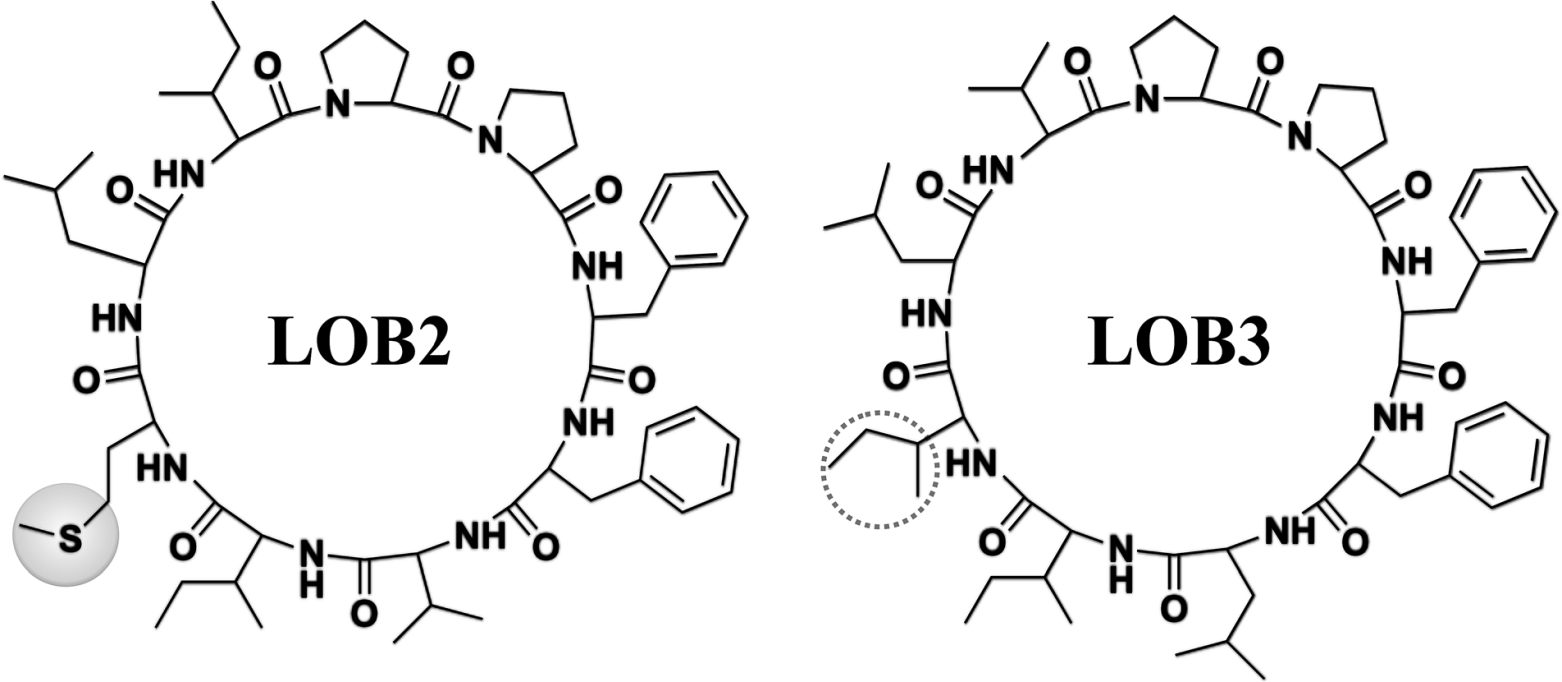
Chemical structures of LOB2 and LOB3.

**Fig. 2:**
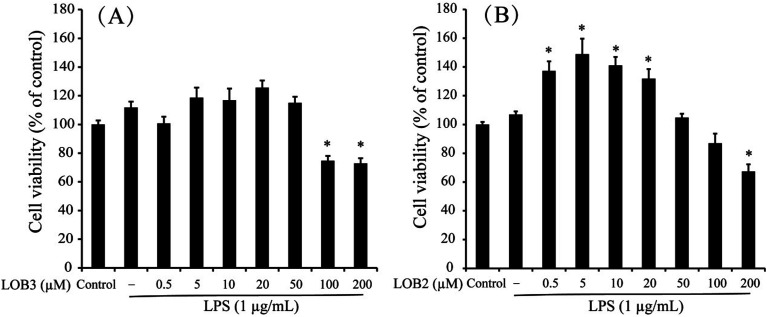
Effects of LOB3 (A) and LOB2 (B) on the viability of THP-1 macrophages determined by MTT assay. THP-1 macrophages were pretreated with various concentrations (0, 0.5, 5, 10, 20, 50, 100 and 200 μM) of LOB3 or LOB2 for 1 h and then the cells were stimulated with LPS (1 μg mL^−1^) for 3 h. Significant differences compared with control group are indicated as *p < 0.05.

The chemical structure of LOB2 and LOB3 was shown in Fig. 1, LOB2 with a methionine residue and LOB3 with an isoleucine residue. An MTT assay was performed to determine LOB2 or LOB3 cytotoxicity to THP-1 cells. LOB3 slightly increased MTT staining at concentrations below 50 μM, but it significantly (*p* < 0.05) decreased cell viability at concentrations higher than 100 μM compared to the control group (Fig. 2A). At concentrations from 0.5–20 μM LOB2 significantly elevated MTT staining while a slightly increased MTT staining was observed at a concentration of 50 μM. When the concentration reached to 200 μM, LOB2 significantly (*p* < 0.05) inhibited MTT staining and presumably reduced cell viability compared to controls (Fig. 2B). These results demonstrated that both LOB2 and LOB3 at concentrations lower than 50 μM did not inhibit the conversion of MTT to its formazan by reduction. Therefore, these concentrations might not induce cytotoxic effects on THP-1 macrophages, thus these concentrations were chosen to study anti-inflammatory activity.

### 3.2 Effects of LOB2 or LOB3 on NO production by LPS-induced THP-1 macrophage inflammation model

**Fig. 3:**
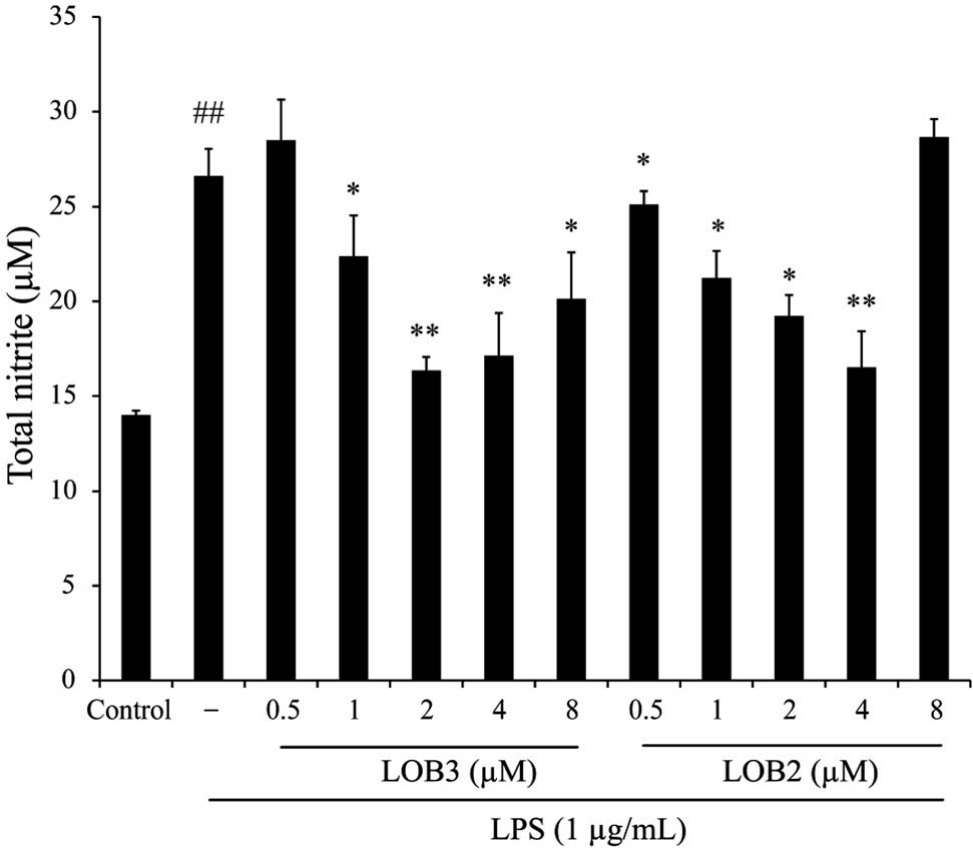
LOB3 and LOB2 prevented the NO production of LPS-induced THP-1 macrophage inflammation model. THP-1 macrophages were pretreated with various concentrations (0, 0.5, 1, 2, 4 and 8 μM) of LOB3 or LOB2 for 1 h and then the cells were stimulated with LPS (1 μg mL^−1^) for 3 h. After treatment, NO production was detected by the Griess reagent. Significant differences among LPS treatments are indicated as *p < 0.05, **p < 0.01. ^##^*p* < 0.01 *versus* the control group.

Inducible NO synthase generates the free radical NO. This pathway is inducible and excess NO production can strengthen the inflammatory response which, can cause severe damage to the host.^22^ Hence, the level of NO accumulation reflects the degree of inflammation and is a useful measure of the effect of therapeutic agents on the inflammatory process. The anti-inflammatory effects of LOB2 and LOB3, were determined on NO accumulation in LPS stimulated THP-1 cells. The synthesis of NO significantly increased to 26.6 ± 1.4 μM (*p* < 0.01) after treatment with LPS alone compared to the vehicle (13.99 ± 0.24 μM; Fig. 3). Whereas, co-treatment with LPS and LOB2 or LOB3 at concentrations of 1, 2 and 4 μM significantly (*p* < 0.05 or *p* < 0.01) reduced NO production. For example, formation of NO decreased to 16.35 ± 0.72 or 19.23 ± 1.10 μM after treatment with 2 μM LOB3 or LOB2, respectively. However, increasing concentrations of the two LOs to 8 μM did not further inhibit NO production.

### 3.3 Effects of LOB2 or LOB3 on LPS-induced release of cytokines in a THP-1 macrophage inflammation model

**Fig. 4:**
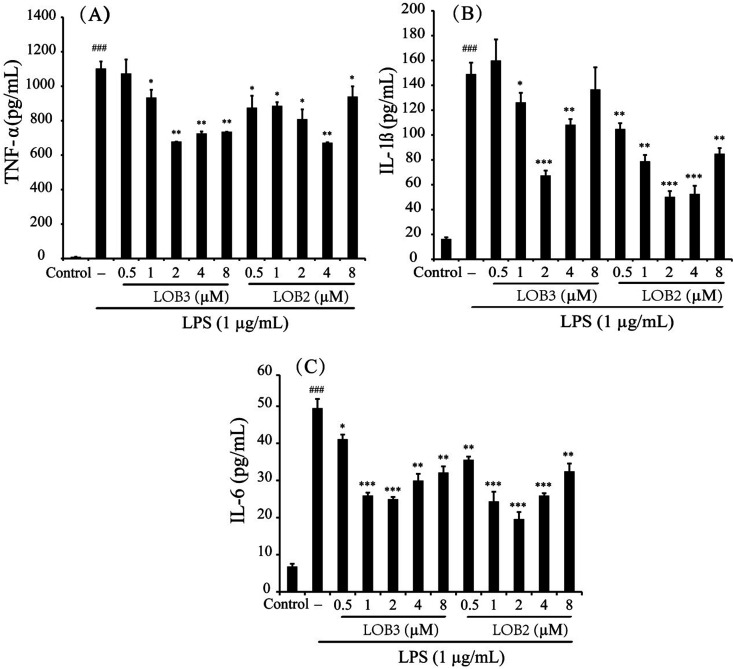
LOB3 and LOB2 inhibited the LPS-stimulated release of TNF-α (A), IL-1β (B) and IL-6 (C) in THP-1 macrophages. THP-1 macrophages were pretreated with different concentrations (0, 0.5, 1, 2, 4 and 8 μM) of LOB3 or LOB2 for 1 h and then the cells were stimulated with LPS (1 μg mL^−1^) for 3 h. After treatment, the production of cytokines was evaluated by corresponding human ELISA kits. Significant differences among LPS treatments are indicated as *p < 0.05, **p < 0.01, ***p < 0.001, ^###^*p* < 0.001 *versus* the control group.

Pro-inflammatory cytokines such as TNF-α, IL-1β and IL-6 are important initiators and enhancers in the occurrence and development of inflammation.^23^ The anti-inflammatory activities of LOB2 or LOB3 were determined by their effects on production of TNF-α, IL-1β, and IL-6 in LPS-stimulated THP-1 cells. In comparison to untreated cells, 3 h of LPS stimulation (1 μg mL^−1^) elicited production of significant amounts of IL-1β and IL-6, and especially of TNF-α (Fig. 4). However, production of LPS-induced cytokines was inhibited by treatment with LOB2 or LOB3. Compared to LPS group, the release of TNF-α decreased to 935 ± 45, 678.8 ± 0.4, 725 ± 11 and 735.7 ± 0.4 pg mL^−1^ (*p* < 0.05 or *p* < 0.01), after treatment with LOB3 (1, 2, 4 and 8 μM, respectively), and significantly (*p* < 0.05 or *p* < 0.01) to 875 ± 69, 886 ± 21, 808 ± 57, 672 ± 2.5, and 940 ± 59 pg mL^−1^, after exposure to LOB2 (0.5, 1, 2, 4 and 8 μM, respectively) (Fig. 4A).

The release of IL-1β in the LPS group was 149.1 ± 9.2 pg mL^−1^, which decreased to 126.2 ± 7.7 (*p* < 0.05), 67.4 ± 3.9 (*p* < 0.001), and 108.3 ± 4.6 pg mL^−1^ (*p* < 0.01) in response to the treatment with 1, 2 and 4 μM LOB3, respectively, and remarkably declined to 104.8 ± 4.6 (*p* < 0.01), 78.8 ± 5.1 (*p* < 0.01), 50.2 ± 4.6 (*p* < 0.001), 52.6 ± 6.5 (*p* < 0.001), and 84.9 ± 4.5 pg mL^−1^ (*p* < 0.01), after cells were treated with 0.5, 1, 2, 4 and 8 μM LOB2, respectively (Fig. 4B). Additionally, LOB2 or LOB3 at concentrations of 0.5–8 μM significantly suppressed secretion of IL-6 (Fig. 4C). Although the concentration range of LOB2 and LOB3 in inhibiting the production of TNF-α, IL-1β, and IL-6 were somewhat inconsistent, concentrations of 1, 2 and 4 μM of each LO suppressed production of the three cytokines. These findings are in accordance with the influence of these compounds on NO production.

### 3.4 Effect of LOB2 and LOB3 on expression of COX-2 in LPS-induced THP-1 macrophage inflammation model

**Fig. 5:**
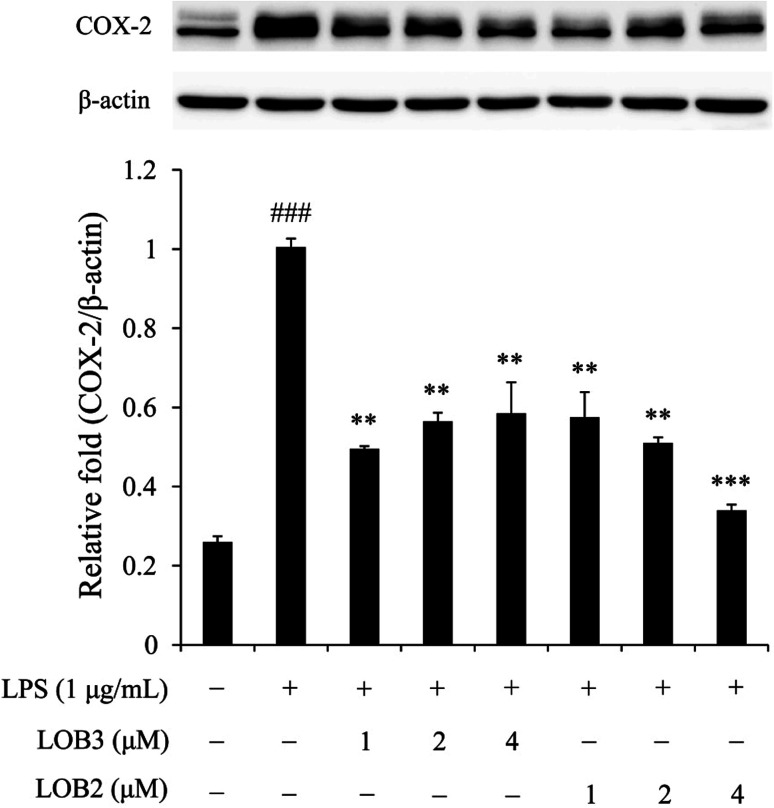
LOB3 and LOB2 suppressed the protein expression of COX-2 in the LPS-stimulated THP-1 cells analyzed by western blot analysis. THP-1 macrophages were pretreated with different concentrations (0, 1, 2 and 4 μM) of LOB3 or LOB2 for 1 h and then the cells were stimulated with LPS (1 μg mL^−1^) for 3 h. COX-2 protein level was corrected to β-actin and was expressed relative to the LPS group (set as 1). ^###^*p* < 0.001 *versus* the control group. Significant differences compared with LPS group are indicated as **p < 0.01, ***p < 0.001.

The enzyme COX-2 is important for synthesis of dienoic eicosanoids. Evidence suggests that COX-2 is involved in the inflammatory process in response to stimulus signals including cytokines and LPS.^24,25^ Therefore, we examined whether LOB2 or LOB3 treatment could affect COX-2 expression in LPS-induced THP-1 cells. As shown using western blot analysis, COX-2 expression was significantly (*p* < 0.001) up-regulated after LPS stimulation compared to controls (Fig. 5). Treatment of cells with LPS plus LOB2 or LOB3 at 1, 2 and 4 μM significantly inhibited COX-2 expression, which was consistent with results showing the inhibition of NO and cytokine secretion by the same treatment.

### 3.5 LOB2 and LOB3 arrested activation of the NF-κB signaling pathway in the LPS-induced THP-1 macrophage inflammation model

**Fig. 6:**
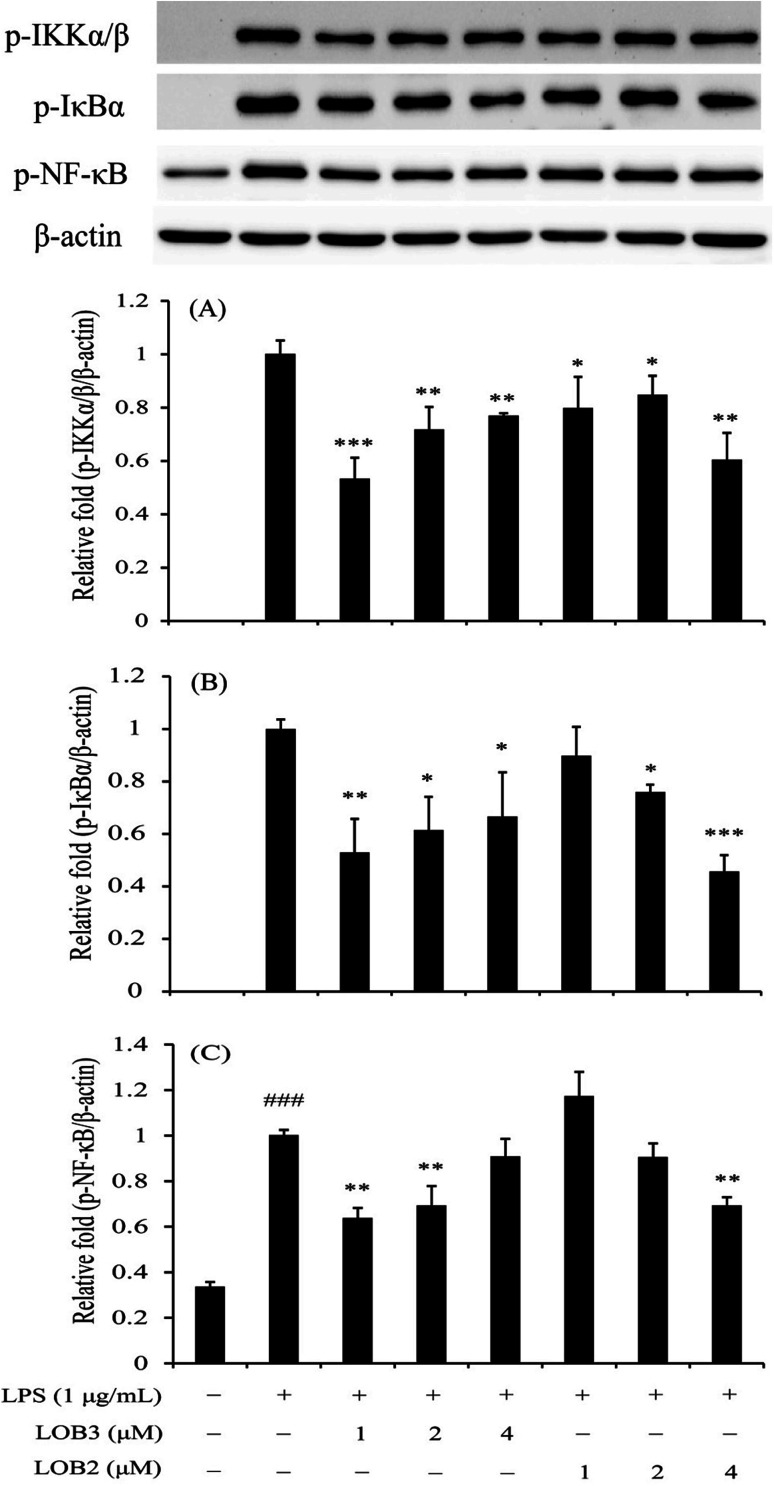
LOB3 and LOB2 inhibited the phosphorylation of IKKα/β (A), IκBα (B) and p65-NF-κB (C) in the LPS-stimulated THP-1 cells evaluated by western blot analysis. THP-1 macrophages were pretreated with different concentrations (0, 1, 2 and 4 μM) of LOB3 or LOB2 for 1 h and then the cells were stimulated with LPS (1 μg mL^−1^) for 3 h. Protein levels of p-IKKα/β, p-IκBα and p-p65-NF-κB were corrected to β-actin and expressed relative to the LPS group (set as 1). ^###^*p* < 0.001 *versus* the control group. Significant differences compared with LPS group are indicated as *p < 0.05, **p < 0.01, ***p < 0.001.

The NF-κB family is a critical signaling pathway that regulates the transcription of numerous pro-inflammatory cytokines (TNF-α, IL-1β, IL-6 and so on), nitrogen intermediates and COX-2 during inflammation.^26,27^ Therefore, this family is an attractive therapeutic target for bioactive compounds. We determined the inhibitory effects of LOB2 or LOB3 on activation of the NF-κB signaling pathway related protein expressions (p-IKKα/β, p-IκBα, and p-p65-NF-κB) in LPS-stimulated THP-1 cells to further explore mechanisms that contribute to the anti-inflammatory activities the LOs. Western blot analysis showed that LPS-induced phosphorylation of IKKα/β was significantly inhibited after treatment with both LOB2 and LOB3 at concentrations of 1, 2 and 4 μM, respectively (Fig. 6). In addition, expression of p-IκBα decreased to 0.53 ± 0.13 (*p* < 0.01), 0.61 ± 0.13 (*p* < 0.05), and 0.67 ± 0.17 (*p* < 0.05), after co-exposure of 1, 2 or 4 μM LOB3 and LPS, respectively, and was down-regulated to 0.76 ± 0.02 (*p* < 0.05) and 0.46 ± 0.06 (*p* < 0.001), after co-exposure to LPS and LOB2 at concentrations of 2 and 4 μM, respectively. Additionally, phosphorylation of p65-NF-κB was attenuated to 0.64 ± 0.04 (*p* < 0.01) and 0.69 ± 0.09 (*p* < 0.01) in the LPS treatment group after treatment with 1 or 2 μM LOB3, respectively. However, phosphorylation was significantly reduced to 0.70 ± 0.04 (*p* < 0.01) only after treating with LPS and 4 μM LOB2. These results suggested that both LOB3 and LOB2 arrested the activation of NF-κB signaling pathway in the LPS-induced THP-1 macrophage inflammation model. Whereas, the active concentration was different, LOB3 at 1 and 2 μM significantly decreased expression of all three phosphorylated proteins induced by LPS, however, LOB2 only exhibited a comparable inhibitory effect at a concentration of 4 μM.

## 4. Discussion

Food-derived peptides not only provide essential nutritional requirements for human growth and development they also are capable of exerting a vast array of beneficial biological actions on human health, either directly in their native form or indirectly following their denaturation through enzymatic hydrolysis and digestion, food fermentation or processing.^28,29^ LOs are a series of hydrophobic orbitides that were first identified by Kaufmann and Tobschirbel,^30^ Morita *et al.*,^31^ and Reaney *et al.*^32^ using amino acid analysis, NMR, MS/MS and IR techniques. Because of their size, unique structural characteristics and complexity, they occupy a chemical “middle space” in drug discovery, thus providing them a broad variety of unusual and potent biological activities, such as immunosuppressive,^33^ antimalarial,^34^ antioxidative,^35^ and anticancer activities.^36^ Furthermore, results from several recent studies show that LOs bind to biomacromolecules and impart *in vitro* antitumor effects on various cancer cell lines based on their different structures.^13,15,16,36^ However, to the best of our knowledge, there is limited literature describing their mode of action as anti-inflammatory agents. Therefore, in this study, we investigated and compared the anti-inflammatory effects of 2 different LOs (LOB3 containing a methionine residue and LOB2 containing isoleucine residue) as well as the underlying effects on the inflammation pathway in THP-1 cells.

Evaluation of recent reports leads to the conclusion that THP-1 monocyte/macrophages are a sensitive, unique and accurate *in vitro* cell model to investigate the mode of anti-inflammatory action of various bioactive products.^21,37,38^ LPS, a component of the Gram-negative bacterial cell wall, is a well-characterized inducer of monocytes and macrophages, which can lead to a cascade of cellular events that ultimately result in the pro-inflammatory response, including the secretion of cytokines and other inflammatory mediators.^39^ Among the cytokines, TNF-α is released early in copious amounts after invasive stimulation and its overproduction is linked to the production of other cytokines such as IL-1β and IL-6 and various inflammatory mediators such as COX-2 and NO that, in turn, regulate gene expression, DNA damage and cell survival.^40–42^ Therefore, assays that reveal changes in cytokine production, NO and/or COX-2 are of great importance in discovering anti-inflammatory natural products and evaluating their mode of action.

In the present study, LPS induced increased secretion of the pro-inflammatory cytokines TNF-α, IL1β and IL-6 as well as NO in THP-1 cells. Whereas, co-treatment of THP-1 cells with LPS and LOB3 or LOB2 at concentrations of 1–4 μM significantly decreased production of the three cytokines and NO, suggesting that both two LOs at similar concentrations are effective inhibitors of LPS-induced pro-inflammatory cytokines and NO production. Increasing the concentration of two LOs to 8 μM did not strengthen but reduced the inhibitory effects of three cytokines and NO to some extent. A similar phenomenon can also be found in other studies. For example, Ishii *et al.*^43^ reported that 100 mg mL^−1^ of glycolipids extracted from spinach inhibited expression of cytokines (IL-6 and MCP-1), adhesion molecules (ICAM-1 and VCAM-1) and p-NF-κB after LPS treatment. The effects were more potent than for treatments with lower glycolipids concentrations (0.1 and 10 mg mL^−1^); furthermore, the glycolipids promote NO production in the presence of LPS. In addition, Lee *et al.*^44^ reported that inhibitory effects of *Schistosoma* egg antigens towards JAK1 at higher concentrations (20 μg mL^−1^) was less effective than at lower concentrations (8, 12 and 16 μg mL^−1^). At concentrations of 1–4 μM, the increased expression of COX-2 in LPS-treated cells was also remarkably suppressed after treatment with the two LOs. It is worth noting that increased concentrations from 1 to 4 μM did not enhance the suppressive effect of COX-2 expression in the LOB3 treated cells, but it was enhanced in the LOB2 treated cells. The phenomena of concentration independent differences in anti-inflammatory responses are possibly due to the immunosuppressive potency of LOB3,^33^ which might cause concomitant decrease in the intensity of the inflammatory response.

NF-κB plays a pivotal role in regulating the survival, activation and differentiation of immune cells exposed to a wide group of stimuli.^45^ As previously reported,^27^ the induction of mediators and most genes involved in the inflammation process could be abolished or attenuated through inactivation of the NF-κB signaling pathway. In this study, we found that LPS activated the NF-κB signaling pathway through up-regulation of p-IKKα/β, p-IκBα and p-p65-NF-κB proteins, which were significantly down-regulated after LPS stimulation by LOB3 at concentrations of 1 and 2 μM or LOB2 at a concentration of 4 μM. When compared to the effective concentration range (1–4 μM) for inhibiting pro-inflammatory cytokines, NO and COX-2, the LOs were less effective at suppressing the NF-κB signaling pathway. This latter result indicates that another signaling pathway might be involved in the observed anti-inflammatory effects and regulated in different degrees by the LOs. Zhao *et al.*^46^ reported that 4-ethylguaiacol from baijiu (a traditional Chinese alcoholic beverage) could inhibit LPS-induced inflammatory responses in THP-1 cells through both NF-κB and MAPK pathways. Overall this study provides substantial evidence that the LOs exhibited modest anti-inflammatory activities by suppressing NF-κB pathway in LPS-stimulated THP-1 macrophages.

The concentration-related effects of LOs in blocking expression of three phosphorylated proteins reflects differences in the response of the signaling pathway and also indicates that LOB3 was a stronger suppressor of the NF-κB signaling pathway. This finding is in agreement with the anticancer effects of LOB3 which is more cytotoxic towards SGC-7901 cancer cells than LOB2.^15^ Almousa *et al.*^47^ studied the anti-inflammatory response of LOB3 and [1–8-NαC],[1-MetO_2_]-linusorb B1 by the evaluation of *trans*-epithelial electrical resistance (TEER) value in Caco-2/RAW-264.7 co-treated cells, and found that LOB3 increased relative TEER values at concentrations greater than 2 μM (∼200% of LPS only wells); in contrast, [1–8-NαC],[1-MetO_2_]-linusorb B1 at a concentration of 200 nM, maintained a higher relative TEER than LPS controls. LOMIX (LOB2, LOB3, [1–8-NαC],[1-(*R*_s_,*S*_s_)-MetO]-linusorb B1, [1–8-NαC],[1,3-(*R*_s_,*S*_s_)-MetO]-linusorb A1, [1–8-NαC],[1-(*R*_s_,*S*_s_)-MetO]-linusorb A2, and [1–8-NαC],[1,3-(*R*_s_,*S*_s_)-MetO]-linusorb A3), derived from flaxseed oil was reported to have excellent *in vivo* therapeutic effects against various inflammatory symptom (acute gastritis, enteritis and hepatitis) using animal models.^14^

Considering that many different mechanisms can be associated with the anti-inflammatory effects of peptides, it is difficult to ascertain specific structural features responsible for LO bioactivity. However, peptide hydrophobicity is a major factor governing the anti-inflammatory response. Many studies have shown that high hydrophobicity in peptides enhances their interaction with cell membranes, this property may aid in modulation of downstream signaling pathways and exhibition of anti-inflammatory effects. For instance, milk casein-derived hydrophobic peptides VPP (Val-Pro-Pro) and IPP (Ile-Pro-Pro), were recently found to exert anti-inflammatory activity *via* the inhibition of NF-κB pathway and reduction of adipokine levels in murine pre-adipocytes.^48^ Apart from hydrophobicity, the cyclic structure and the presence of Pro amino acid also play a crucial role in bioactivity. It has been reported that peptides with cyclic structure and/or Pro are rigid, less prone to proteolytic digestion, easily penetrate membranes and are absorbed in the intestine when consumed orally. These properties contribute to bioactivity including anti-inflammatory activity.^49,50^ Wu *et al.*^51^ reported that two novel cyclic peptides with Pro, fanlizhicyclopeptide A [*cyclo*(Pro-Pro-Tyr-Leu-Pro-Gly-Val)] and fanlizhicyclopeptide B [*cyclo*(Pro-Ile-Tyr-Ala-Gly)] obtained from *Annona squamosa* fruit, exhibited *in vitro* anti-inflammatory effects on the inhibitory release of TNF-α and IL-6 in LPS-induced RAW 264.7 macrophages. Therefore, the cyclic structure, high hydrophobicity and presence of Pro amino acid residues in LOB2 and LOB3 might contribute their anti-inflammatory properties. Further research is required to elucidate their roles.

## 5. Conclusions

This study shows that the *in vitro* anti-inflammatory activity of LOs in the LPS-induced THP-1 macrophage inflammation model occurs through suppression of pro-inflammatory cytokines as well as the downregulation of the NF-κB pathway. In addition to inhibitory effects of the LOs towards the release of cytokines and NO and protein expression of COX-2 were comparable to some extent and the concentration of action was limited to the range of 1–4 μM. Interestingly, their concentrations in suppressing the NF-κB pathway were different. Concentrations of 1 and 2 μM LOB3 could downregulate the NF-κB pathway; however, LOB2 was comparably less effective where a concentration of 4 μM was required for inhibition of the NF-κB pathway. Results from this study suggested that LOs modestly suppress NF-kB pathway and only at a very narrow dose range.

## Abbreviations

LPS: Lipopolysaccharide
ROS: Reactive oxygen species
IL: Interleukin
TNF-α: Tumor necrosis factor alpha
LOs: Linusorbs
LOB2: [1–9-NαC]-linusorb B2
LOB3: [1–9-NαC]-linusorb B3
IKK: IκB-kinases complex
NO: Nitric oxide
Met: Methionine
MetO: Methionine *S*-oxide
RPMI: Roswell Park Memorial Institute
FBS: Fetal bovine serum
MTT: 3-(4,5-Dimethylthiazol-2-yl)-2,5-diphenyltetrazolium bromide
DMSO: Dimethylsulfoxide
PBS: Phosphate buffered saline solution
PMA: Phorbol 12-myristate 13-acetate
RIPA: Radio immunoprecipitation assay
ELISA: Enzyme-linked immunosorbent assay
COX-2: Cyclooxygenase-2

## Conflicts of interest

There are no conflicts of interest to declare. Dr Martin J. T. Reaney is the founder of, and has an equity interest in, PTD (Saskatoon, SK, Canada: previous company name is Prairie Tide Chemicals Inc.). Dr Youn Young Shim is a Market Consultant for PTD. The terms of this arrangement have been reviewed and approved by the University of Saskatchewan (Saskatoon, SK, Canada) in accordance with its conflict of interest policies.

## Supplementary Material
